# Design of Occupational Therapy Interventions for Middle-Aged and Elderly Family Caregivers

**DOI:** 10.3390/healthcare9030275

**Published:** 2021-03-03

**Authors:** Wen-Huei Chou, Ya-Ling Ko, Xiang-Yu Huang

**Affiliations:** 1 Department of Digital Media Design, National Yunlin University of Science and Technology, Yunlin 64002, Taiwan; cris@gemail.yuntech.edu.tw; 2 Graduate School of Design, National Yunlin University of Science and Technology, Yunlin 64002, Taiwan; D10630001@gemail.yuntech.edu.tw

**Keywords:** narrative therapy, occupational therapy, life review, interaction design, caregiver

## Abstract

This study aimed to develop an interactive app for occupational therapy interventions for middle-aged and elderly family caregivers by integrating life review and narrative therapy. The results indicated that the interventions improved the mood of individuals, but the improvement in the quality of life was less significant due to the multiple facets of life. The interface design of the interactive app had good operating characteristics and was above average in terms of learnability and usability. Overall, the intervention program positively improved participants’ psychological aspect, which was in line with the purpose of the life review. Thus, a focus group put forward specific suggestions on the contextual construction of life review, the intelligent development of guiding questions, scenario simulation, and the introduction of creative collaboration functions, which could be used as a reference for subsequent content adjustment and interface design.

## 1. Introduction

Statistics from the World Health Organization (WHO) in 2018 suggest that more than 15% of the world’s population has some form of disability, which is increasing with the years due to the population aging and chronic diseases [1]. Some people with disabilities need to seek care support to maintain their daily lives. According to 2016 Taiwan’s Ministry of Health and Welfare (MOHW), the demand for long-term care (LTC) in Taiwan is projected to exceed 800,000 people by 2020 [2]; however, in 2018 the Primary Family Caregiver Report indicated that the numbers of hours and average years of care increase with the age of caregivers [3]. As a result, the stress load on caregivers increases with years of care.

With aging, declining birthrate, and changes in family structure, it is more common to see elderly caregivers taking care of older people, which puts increasing pressure on family caregivers and makes them the main care providers in the LTC system. In addition, recent studies also have demonstrated that women are mostly unpaid caregivers of informal care in the family [4,5]. Gallagher et al. suggested that most families provided a certain degree of care support for the disabled but also faced health and safety issues, and they mentioned that caregivers were sometimes referred to as “secondary caregivers” [6]. Studies have suggested that chronic caregiving stress easily leads to physical and psychological issues in caregivers and affects caregiver health and caregiving outcomes, which results in increased mortality rates among those cared for [7,8]. 

As a result, numerous studies have examined the impact of caregiver stress and caregiving load on the quality of life; and includes focusing on the multiple health problems that the stress of care may result in the individual [9,10]. Research has further demonstrated that caregivers often face multiple challenges such as the lack of knowledge and caregiving abilities for diseases, weakened economic and social ties, and negative physical and psychological dilemmas [11,12]. Caregivers face diverse caregiving situations, and multiple sequences of events add to the complexity [10], complicating the theoretical development of relevant research.

In 1946, The United Nations prepared the “Constitution of the World Health Organization”, and explicitly defined health as a state of physical, mental, and social well-being completely, not only the absence of disease or weakness [13]. However, the negative impact of caregiving stress and excessive mental load causes worsening health of the family caregiver. Since lifestyle changes and weakened social connections tend to neglect psychological load, remove the opportunity to seek immediate help, and develop subtle stress issues over time [14]. Some studies have revealed caregivers can improve their health and quality of life by reducing internal stress and enhancing the self-efficacy of individuals and further enhance the quality of care [15,16,17]. Therefore, it’s crucial to provide psychological support. Although there is some support strategy for psychological counseling and respite care, the utilization rate is still insufficient [18,19]. The reason for the insufficiency includes the fact that the caregivers are not leaving the person who is cared for, or time and space being restricted leading to use of support services difficulties [20,21].

Graff et al.’s studies indicated that the design and implementation of occupational therapy can improve daily performance, communication, and quality of life of caregivers [22]. Life review is a method of narrative therapy and is often applied to psychotherapy. The object of implementation has also moved on from older adults to expand its scope of application to include different age groups [23,24,25,26]. The clinical application of narrative therapy focuses on meaning-making, allowing the individual to interpret his/her own problems through dialogue. It also allows for the clarification of previously ambiguous experiences as well as the organizing and understanding of complex and chaotic states [27,28]. Researchers and their objects explore the implications of content through an examination of the entire narrative process [29,30]. Transformation enables old experiences to take on new meanings and connect with current behaviors, whilst negative memories of the past are given new meanings in the process [27,31] to enhance an individual’s self-identity.

The rise of digital technologies provides a new type of auxiliary tool for various treatment options. The World Health Assembly (WHA) in 2018 agreed that digital technologies play a key role in improving public health and encouraged their application in the health sector [32]. The development of the internet has provided effective applications that can enhance social support and the quality of life of caregivers [17,33]. Therefore, more and more innovative solutions become the focus of the discussion. Barbabella et al. developed the web platform of family caregivers and called it the “InformCare”. It does not only provide some information of care but also builds the social network (chat room, video, etc.) [34]. Mobile communication software could address different perceptual needs of family members, affecting the perceived usefulness and perceived entertainment among middle-aged and adults [35]. Internet of Things (IoT) and technology applications (such as virtual reality, VR) are implementing human-centered operation functions actively, and help people readapt and maintain their daily life [36,37]. Therefore, integrating innovative technology to support intervention program design has become the development trend.

As mentioned above, this study adopted digital tools as the carrier of design applications and integrated the theoretical foundations of life review and narrative therapy for interactive application program curriculum planning, design, and implementation. The target object was middle-aged and elderly caregivers with more than half a year of caregiving experience and exploring the impacts involved, including psychological, physical, and social relationship before and after implementation of an intervention program. This may provide therapists with the opportunity to assist participants in reviewing their life course, transforming negative emotions, and promoting their psychological health while providing occupational therapy. Secondly, evaluate the usability of digital tools and examine the operation of the interactive app. Through focusing on usage effectiveness, the interactive app as an intervention program will be able to reduce negative physical and psychological dilemmas, while enhancing the quality of life.

## 2. Materials and Methods

This study combines theories including narrative therapy and life review as the basis of application content design and intervention program development. The study adopted the triangulation method to evaluate the rationality of theories, methods, and data collection and analysis, and to ensure the consistency of research results. The design of the research methodology, expert interviews, effectiveness evaluation of case interventions, and focus group discussions were planned and implemented to establish the design results of this study, thereby meeting the research objectives.

### 2.1. Expert Interviews

Based on design requirements, the principles of the three-pronged approach to building an interactive design of life review were applied as follows: (1) Construct the main theories and implementation requirements of life review activities; (2) Deconstruct the singularity of life review stories through systematic dialogue and guidance and assist in the reconstruction process; and (3) Through practical operation and discussion of needs, understand the problems faced by the target population and provide feedback on the needs of the interactive design and its application to future implementation.

In response to the participants’ needs in this study and the use of life review for the interactive design study, the interviewers should be professional, including psychological counselors, experts in life review guidance, and occupational therapists (with five years of clinical experience), to build a theoretical foundation and introduce practical experience as the basis for research development. This study had two phases. In Phase I, semi-structured interviews were conducted with psychological counselors and experts in life review guidance, covering the following research areas to establish the implementation framework of the life review method as well as the current status and key elements of intervention implementation.

The planning of interview topics included topic guidance and operational design during the implementation of life review, setting up the research participants and implementation requirements, and post-implementation effectiveness evaluation. Elements corresponding to the research focus were presented after the interview as a reference for subsequent planning of the interaction design and as suggestions for guidance and operation of clinical implementation (Table 1).

Phase II focused on the initial development of the unit topics of life review through an interview with the occupational therapist and further established the functional requirements of the interactive interface for back-end application in clinical trials.

Bulter introduced the concept of life review as a therapeutic concept; he even argued that it is an effective means to promote the growth of an individual [38,39]. The use of life review as a therapeutic approach prioritizes the time sequence of important events in an individual’s life. In this process, creating new narratives aids individuals in focusing on the strengths and wisdom they possess with age, rather than their weaknesses [40]. Savickas established a framework for the implementation of career review as an intervention by exploring practical procedures involving five elements: (1) Construction: assisting individuals in detaching themselves from their stories and reinterpreting their experiences; (2) Deconstruction: starting with small stories and assisting individuals in constructing new experiences and interpretations of the stories; (3) Reconstruction: assisting in transforming small events into components that construct thematic values to form a larger story. The process reorganizes the meaning of the individual and his/her relevance to society; (4) Co-construction: after guiding the individual to reconstruct his/her own micro-history, further exploration helps to resolve the conflicts of the past and opens up new opportunities; (5) Action: the achievements of the past in retrospect and the uncertainties of the present in life are connected to the future through positive actions [41]. As mentioned above, these five elements will serve as the basis for application program content design.

The purpose of the review was to guide the individual through a narrative process of re-definition, deconstruction, and reconstruction, and also enable them to rebuild self-meaning, thereby regulating oneself and improving one’s relationship with others. Therefore, the topic design emphasized the universal life experience and the relationship between middle-aged and elderly caregivers, the cared for, and families. The main topic design was centered around the core of life review, and with several open-ended units under the topics (Table 2).

### 2.2. Design and System Development of Life Review Interactive App

This study used “prototyping” as a form of system planning and development. Its advantages are that it can be used for low-cost and rapid design in a limited time and be directly applied to the user terminal for communication, testing, and real-time feedback and correction. The selection of digital tools was based on the needs of the subjects. A 12.9-inch hand-held tablet personal computer (tablet PC) was used as the development device, Mac OS was used as the main operating system for program development, and the development environment was established with Xcode. Swift programming language was adopted which has high integrity combined with Xcode. Additionally, considering the limitations of the interface for the older person, colors, interface, and hierarchical function menus were designed to be highly recognizable and intuitive to operate to reduce the burden of use.

**Figure 1:**
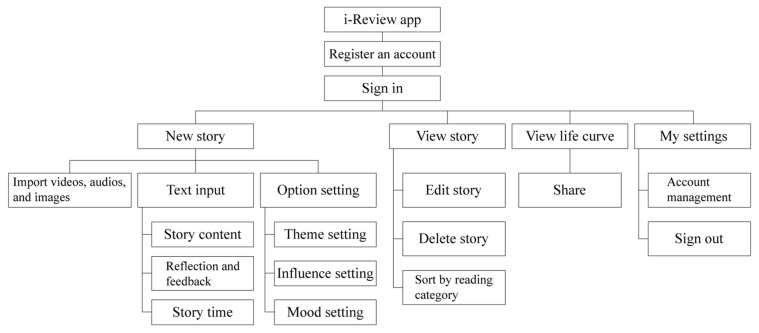
Functional structure of the i-Review interactive app.

**Figure 2:**
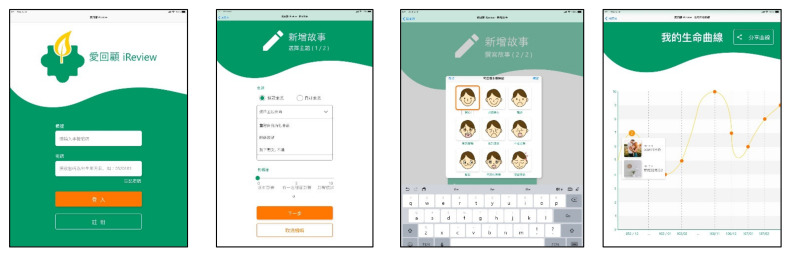
The interface of the i-Review interactive app.

The life review interactive app was named “i-Review”, whose design was introduced in the key points in the previous section for preliminary functional planning. The requirements of the interactive app were summarized as follows: (1) Implementation method of life review: introduce unit topics to assist the subject in the review; (2) Influence of life events on the subject: the level of influence, the time of the event, and the emotional response to the event; (3) Application of auxiliary tools: provide basic functions such as drawing, image, and audio-visual import; (4) Re-experience events and transform them into new viewpoints: provide functions such as viewing, adding, deleting, and modifying usage records; and (5) Review the timeline to understand the cause and effect of events: link past records to create a life story graph. Based on the above five points, we proposed the functional architecture of the system and interface design as shown in Figure 1 and Figure 2.

### 2.3. Experiment and Operation Process Design

The experiment lasted four months and five days. Subject to the operation procedure, the experiment was first guided by a functional therapist, who selected the participants for pre- and post-tests; an interactive app was used as an auxiliary tool to implement intervention activities. A case evaluation report was completed after the experiment to examine the effectiveness of the intervention. Since the course involved privacy, the research team only acted as an observer at this stage. The second step was to evaluate whether the app was effective in helping the participants to complete their life review and obtain information on the interface operation terminal as a basis for modifying the design.

Participants were selected by therapists from caregivers of outpatients through intentional sampling and signed the informed consent form prior to the implementation of this study. The age of the target population was based on statistics of MOHW in 2017 and the Taiwan Association of Family Caregiver in 2007 which presented that more than 60% of family caregivers are between 40 and 65 years old and belong to the middle-aged group [3,42]. After understanding the purpose of the intervention course and the implementation method, the therapists conducted a psychological intensity test using the “Taiwanese Depression Questionnaire” developed by the John Tung Foundation [43]. The content of the questionnaire was 18 questions and is mainly to evaluate the feeling of the individual in the past week with a 4-point Likert Scale as the measure. The main target group was those whose total score fell between 9 and 18 (a score below 9 indicated a normal psychological condition, whereas a score above 18 indicated a high risk-group). A total of 12 participants aged between 40 and 68 years were recruited, including 8 women and 4 men. The most common relationship with the cared-for was husband or wife, but mainly wife. Additionally, four participants resigned from their job due to taking on care responsibilities. Baseline data are shown in Table 3.

The intervention implementation plan asked each participant to complete four life review activities with different unit topics each time in one hour, which were conducted in the treatment room of the branch hospitals.

Data on effectiveness evaluation were divided into three parts: (1) Records of implementing the intervention program: the therapist-assisted in recording the implementation process, including the observation of the planning of unit topics and the use of interactive design; (2) Questionnaire: the Beck Depression Inventory-II (BDI-II), World Health Organization Quality of Life-BREF (WHOQOL-BREF) Taiwan version and System Usability Scale (SUS) were used; and (3) Expert focus group interview: a group composed of the research team, occupational therapists, and psychological counselors made suggestions on the implementation feedback of the intervention course, the participants’ operation behaviors, and the appropriateness of content design, in which process de-identification was adopted to protect the privacy of participants.

## 3. Results

The study results are presented in two phases. In Phase I, scales were used to evaluate the effectiveness of the intervention program and the usability of the interface; in Phase II, a focus group discussion was held to draw objective conclusions. SPSS 25.0 (IBM, Armonk, NY, USA) for Windows was used for descriptive analysis of the scale results.

In Phase I, three scales were applied to evaluate the implementation effectiveness. BDI-II second edition was revised by Beck et al. in 1996 [44], and translated into Chinese by Chen in 2000 [45]. The scale showing high reliability [46] and was used to identify whether the intervention achieved emotional improvement in the target population. There were 21 questions, with a scale of 0 to 3 points for each question. Participants, through self-assessment, expressed their feelings over the past two weeks and answer to the scale. The acceptable range was 0–13 points, 14–19 points indicated mild depression, 20–28 points indicated moderate depression, and 29–63 points indicated severe depression. Table 4 presents the pre-test and post-test data of Wilcoxon Signed Ranked.

After the pre- and post-tests, the mean score decreased from 11.92 to 8.83—an overall decrease of 3.09—and the test results reached a significant difference (*p* = 0.038 < 0.05). Although the means measured in the pre and post-tests were within the acceptable range of 0–13, it was difficult to conduct a further investigation because of the limited sample size, length of caregiving, and access to intervention reports.

Regarding whether the intervention could improve the quality of life of the target population, the WHOQOL-BREF Taiwan version was used to conduct measurement and analysis [47]. Four main domains comprising 28 items were used to measure the overall quality of life and health status, involving physical, psychological, social relationship, and the environment. There were two additional items in the Taiwan version, using a 5-point Likert scale as the measure. The points of test data were totaled through reverse items, where a higher total score indicated higher satisfaction with the quality of life.

The overall quality of life was not significantly affected by the intervention (physical *p* = 0.656 > 0.05; psychological *p* = 0.07 > 0.05; social relationship *p* = 0.157 > 0.05; environment *p* = 0.178 > 0.05), but the pre and post-test means increased slightly, with the psychological domain being the most obvious. Item 26 mainly investigated the negative feeling of the individual, and the results after conversion show significant differences (*p* = 0.034 < 0.05). It means the participant’s emotional feelings have improved after the implementation of an intervention program (Appendix A, Table A1 and Table A2).

Finally, the System Usability Scale (SUS) and interviews were used for satisfaction verification and operational feedback after the end of the entire experiment, hoping to understand the usability and user mobility of this APP in terms of the effectiveness of the interactive design, so as to serve as a reference for future design improvements and applications in similar interactive design applications in intervention activities. SUS, which is widely applied in the perception of the overall use of systems and products, is associated with usability and learnability. There were 10 questions, measured using a 5-point Likert scale. The scale was designed with positive and negative questions, which were calculated by subtracting 1 point from the original score for positive questions and 5 points from the original score for negative questions. Based on previous findings, there were 2 questions on learnability and 8 questions on usability, and the original scores required conversion and multiplication to obtain a two-faceted system usability score (0–100) [48]. For the definition of the score benchmark, the mean score of 70 on the scale proposed by Bangor et al. was employed as the test benchmark [49]. Table 5 shows the evaluation and test results.

The overall mean scale score was 74.4; the scores for learnability and usability after conversion were 73.9 and 73.7, respectively, which were above the mean score. This indicated that the developed interactive app was with good operability on the system interface. Besides, during the interface evaluation process, the subjects were asked about the experience of the interface operation. They gave positive comments on the usability, smoothness, stability, and color settings, while improvements were needed on option locations, text input, and content settings.

During Phase II, focus group discussions were held to evaluate the overall effectiveness of the program before and after the intervention. The content design and feedback on the use of the interactive app as a guide were discussed, and the results of analyzing Phase I were incorporated. All the discussions were held online. The following results emerged from the focus group discussions on the operation and results of the intervention.

1. The initial design of unit topics failed to fully reflect the life cycle and development of an individual, and its meaning was weakened.
2. Life review focused on the improvement in psychology, and the improvement in the quality of life could not be proved through the intervention of life review.
3. The key factor for significant psychological improvements was the trust between the subject and the therapist, and the appropriateness of the human guidance and unit topics affected the depth of the individual’s narrative.
4. The implementation of the intervention program often involved the time when the cared-for had a return visit, and the subjects were unable to grasp the situation of the cared-for immediately, resulting in decreased concentration.
5. The app interface was not difficult to operate, but there remained some digital disparities in the target population and they needed external assistance to guide the operation.
6. Relying on the built-in materials of the system for operation, the target population lacked the incentive to import the real event content, which also affected the depth of the narrative.

Based on the above results, experts have made suggestions on the design and improvement of interactive apps in future research. Although unit topics covered the overall life history after experts’ evaluation, the design of the app can further help individuals to connect their key events, which induces more stories and deepens the understanding of their own through contextual construction, thereby achieving effective integration. The measurement results of the system interface showed its learnability and usability; however, at this stage, it remains essential to rely on the physical guidance for the interpretation of experiences and acts of reflection during the operation and establishment of the system. To reduce time and space constraints, the system can be operated independently, a guideline and question structure for each unit can be developed, and contextual design can be included at an appropriate time in the timeline of key events. To enhance the willingness to use, the system can be designed in the form of e-books, providing functions of freewriting, template editing, and post-production, as well as review and collection, including output in the form of music matching. A creative operation mode can increase the fun of operation and enhance the user’s willingness to operate.

## 4. Discussion and Conclusions

Through the design and implementation of an interactive app, this study aimed to provide therapists with guidance and assistive tools for life review and to understand the impact of life review activities as an intervention program on middle-aged and elderly family caregivers, especially at a psychological and quality of life level.

Reflecting the implementation framework of the career review proposed by Savickas in 2012 [41], this study assisted individuals in describing and interpreting their life experiences through the guidance of a therapist, and constructed short stories using unit topics, which agreed with the two connotations of “construction” and “deconstruction.”

From the results of the BDI-II analysis, the implementation of the intervention program was beneficial to the psychological improvement of participants, which was in line with the purpose of life review. The usability analysis of the interactive app revealed that the system interface had good operational functions. Nevertheless, the improvement in the quality of life had no significant difference and only exhibited significant differences in item 26, which investigated the negative feelings of an individual. As experts have stated, many variables affect the quality of life, and it is difficult to improve an individual’s quality of life immediately through the intervention program of life review. It is suggested that future evaluations continue to prioritize the individual’s psychological stability, and multi-level discussions can be conducted through long-term experimental observations and the age distribution of the caregivers. Furthermore, there is evidence that the proportion of women as caregivers is significantly higher than men [4,5]. While reflecting the gender ratio of the participants in this research, this also creates a new issue at the same time, that is whether the implementation of the intervention program can have a positive impact on a user of a different gender. According to our results, life review activities as an intervention program can improve the psychological health of middle-aged and elderly family caregivers, and digital technologies as clinical tools are feasible and suitable for occupational therapy.

This study collected the user’s operation mode and conducted pre- and post-experiment interviews with experts and scholars to compile the suggestions for further improvements on the design: (1) strengthen the timeline function: linking the nodes of key events and recording the impact of the events on the individual to help sort out and unify the life cycle; (2) incorporate the guidance function: the future interface function can include the design of the framework of guidance words, construct an intelligent guidance mechanism, and provide users with a more convenient and immediate feedback operation mode; (3) digitalize life stories: the design of e-books provides creative functions and enriches the meaning of stories. In addition to the above three suggestions, this study also proposed a fourth suggestion with reference to the co-construction concept mentioned by Savickas [41]: (4) develop collaborative functions to encourage the extension of life stories from the individual to the family, and enrich life stories through the review and documentation of multiple perspectives. A process of co-participation in creation can also be formed to specifically construct new values for the family and significant others [50], contributing to creating stronger bonds with the family and society.

## Figures and Tables

**Table 1:** Research focus and key elements for the implementation of life review activities.

Research Focus	Key Aspects	Key Elements
1. Establish the implementation structure of the life review method	1. Core aspect of the life review method	1. Affect the psychological conditions of the target group and achieve self-integration; 2. Confirm the goal and direction of guidance. 3. Middle-aged and older participants are more suitable as target participants than those in other stages. Learning and reflection during this stage help to strengthen the individual’s self-integration as he/she moves towards old age.
	2. Key factors affecting the conduct of life review	1. Physical and psychological assessment between the activity facilitator and the target group; 2. The relationship of trust between the activity facilitator and the target group.
	3. Basis for evaluation of the effectiveness of implementing life review activities	1. After guidance, reconsider one’s own role and life experience, reconcile with oneself, and develop new perceptions and perspectives to guide the individual to face the future; 2. The psychological condition of the target group is the key to effectiveness; 3. Set up appropriate indicators to evaluate the difference before and after the intervention of life review activities.
2. Implementation status and essentials of intervention activities	1. Guiding method of life review	1. Emphasis is placed on the participants’ narration of profound experiences (which may be non-linear) that have influenced them; 2. Taking into account the individual differences of the target group, the facilitator should adjust the facilitation content timely; 3. Assist the target group in learning to reconcile with the pain and suffering caused by negative experiences.
	2. Operation and precautions for life review	1. It is recommended to conduct the activity in the morning and each activity should not exceed 2 h; 2. Activities should be conducted in a separate and undisturbed space.
	3. Combine the operating effects of different aids	Different from traditional hand-written works and drawings on paper, life review guidance and operation mode are translated and applied with the aid of digital technologies to avoid time and space limitations.

**Table 2:** Topic design and content of life review units.

Main Topic	Unit Topic	Content
1. Rebuild the meaning of self-role	1. The most memorable growth experience	Describe the most influential people and things in your growth process
	2. The turning point in your life	Talk about the highs and lows in your life course
2. Relationship integration	1. Family memoirs	Reminiscence of your family in photos, images, and paintings
	2. My caregiving journey	Describe your relationship with the cared for and how it happened.
	3. Satisfaction puzzle	Imagine what elements of life can be put together to satisfy yourself?
3. Release conflicts and dissatisfaction	1. The lost beauty	Describe an event in your life that you regret? How would the same event be repaired and changed if it were to happen again?
	2. The happiness equation	Describe the memories of your life that make you feel happy
	3. My dream house	Describe a vision of your future and the goals you want to achieve

**Table 3:** Baseline data of participants.

No	Sex	Age (Years)	Relationship with the Cared for	Disease of the Cared for	Length of Caregiving (Years)	Employment Status of Caregivers	
						Before Caregiving	After Caregiving
1	M	48	Mother and son	Stroke	8	Employed	Unemployed
2	F	62	Ex-wife	Stroke	1	Employed	To be employed
3	M	58	Husband and wife	Stroke	5	Employed	Unemployed
4	F	68	Husband and wife	Brain injury	4	Retired	Retired
5	F	40	Husband and wife	Stroke	2	Employed	To be employed
6	M	48	Mother and son	Myelopathy	1	Employed	Unemployed
7	M	68	Husband and wife	Stroke	4	Retired	Retired
8	F	41	Husband and wife	Stroke	3	Unemployed	Unemployed
9	F	49	Husband and wife	Stroke	2	Employed	Unemployed
10	F	48	Mother-in-law/daughter-in-law	Stroke	1	Employed	To be employed
11	F	40	Mother and daughter	Carbon monoxide poisoning	1	Employed	To be employed
12	F	56	Mother and son	Cerebral palsy	20	Unemployed	Unemployed

**Table 4:** Wilcoxon signed ranked test with Baker Depression Inventory-II (BDI-II) Second Edition.

N	Pre-Test		Post-Test		Paired Samples *t*-Test	
	M	SE	M	SE	Z	*p*-Value
12	11.92	10.352	8.83	7.907	−2.075	0.038 *

* *p*-value < 0.05.

**Table 5:** Evaluation and test results of the interactive app using System Usability Scale (SUS).

N	Evaluation Index	M	Max.	Min.
12	Total scale score	74.4	90	62.5
	Total learnability score	73.9	100	37.5
	Total usability score	73.7	90.625	62.5

## Data Availability

Written informed consent has been obtained from the participants to publish this paper.

## Appendix A

**Table A1:** Wilcoxon signed ranked Test with WHOQOL-BREF Taiwan version items (N = 12).

Domain	Item		Pre-Test		Post-Test		*p*-Value
			M	SE	S	SE	
overall	1	General QOL	3.17	0.72	3.25	0.75	0.564
	2	General health	3.25	0.62	3.33	0.78	0.564
Physical	3	Pain and discomfort *	3.83	0.83	4.33	0.78	0.190
	4	Dependence on medicinal substances and medical aids *	4.50	0.52	4.50	0.67	1.000
	10	Energy and fatigue	3.25	0.62	3.33	0.65	0.705
	15	Mobility	3.42	1.24	3.42	1.24	0.915
	16	Sleep and rest	2.92	0.90	3.25	0.97	0.234
	17	Activities of daily living	3.50	0.67	3.42	1.00	0.763
	18	Work capacity	3.50	0.67	3.50	0.52	1.000
psychological	5	Positive feelings	2.08	0.79	2.42	0.90	0.206
	6	Spirituality, religion and personal beliefs	3.08	1.00	3.25	0.75	0.480
	7	Thinking, learning, memory, and concentration	2.75	0.75	3.00	0.85	0.366
	11	Bodily image and appearance	3.08	0.51	3.17	0.58	0.564
	19	Self-esteem	3.33	0.65	3.33	0.65	1.000
	26	Negative feelings *	3.17	0.94	3.67	0.65	0.034 *
social relationship	20	Personal relationships	3.50	0.80	3.58	0.67	0.655
	21	Sexual activity	3.17	0.94	3.42	0.67	0.180
	22	Social support	3.75	0.87	3.50	0.90	0.257
	27	Being Respected	3.33	0.89	2.91	0.79	0.025 *
Environment	8	Physical safety and security	3.08	1.08	3.17	0.83	0.655
	9	Physical environment	3.50	0.52	3.50	0.52	1.000
	12	Financial resources	2.83	1.27	3.08	1.00	0.317
	13	Information and skills	3.17	0.72	3.25	0.97	0.655
	14	leisure activities	2.17	0.94	2.83	1.03	0.071
	23	Home environment	3.50	0.67	3.42	0.90	0.705
	24	health service	3.83	0.58	3.92	0.79	0.655
	25	Transportation	3.83	0.72	3.67	0.49	0.317
	28	Eating	3.42	1.16	3.58	1.00	0.608

item * = reverse coding. * *p*-value < 0.05.

**Table A2:** Wilcoxon Signed Ranked Test of Domains in Pre- and Post-tests.

Domain	Pre-Test		Post-Test		*p*-Value
	M	SE	S	SE	
Physical	14.24	1.81	14.71	2.13	0.656
psychological	11.67	2.28	12.56	1.97	0.070
social relationship	13.75	2.14	13.42	1.88	0.157
Environment	13.04	2.28	13.52	2.25	0.178
